# Wnt5 signaling in vertebrate pancreas development

**DOI:** 10.1186/1741-7007-3-23

**Published:** 2005-10-24

**Authors:** Hyon J Kim, Jack R Schleiffarth, Jose Jessurun, Saulius Sumanas, Anna Petryk, Shuo Lin, Stephen C Ekker

**Affiliations:** 1 Department of Genetics, Cell Biology, and Development, University of Minnesota, Minneapolis, MN 55455 USA; 2 Department of Molecular, Cellular, and Developmental Biology, University of California, Los Angeles, Los Angeles, CA 90095 USA; 3 Department of Pediatrics, University of Minnesota, Minneapolis, MN 55455 USA; 4 Department of Laboratory Medicine and Pathology, University of Minnesota, Minneapolis, MN 55455 USA

## Abstract

**Background:**

Signaling by the Wnt family of secreted glycoproteins through their receptors, the frizzled (Fz) family of seven-pass transmembrane proteins, is critical for numerous cell fate and tissue polarity decisions during development.

**Results:**

We report a novel role of Wnt signaling in organogenesis using the formation of the islet during pancreatic development as a model tissue. We used the advantages of the zebrafish to visualize and document this process in living embryos and demonstrated that *insulin*-positive cells actively migrate to form an islet. We used morpholinos (MOs), sequence-specific translational inhibitors, and time-lapse imaging analysis to show that the Wnt-5 ligand and the Fz-2 receptor are required for proper *insulin*-cell migration in zebrafish. Histological analyses of islets in *Wnt5a*^-/- ^mouse embryos showed that Wnt5a signaling is also critical for murine pancreatic *insulin*-cell migration.

**Conclusion:**

Our results implicate a conserved role of a Wnt5/Fz2 signaling pathway in islet formation during pancreatic development. This study opens the door for further investigation into a role of Wnt signaling in vertebrate organ development and disease.

## Background

Wnt signaling pathways play important roles in both normal development and in the pathogenesis of a variety of diseases, including cancer [1]. Activation of a Wnt signaling pathway requires interaction between a secreted glycoprotein, Wnt, and a seven-pass transmembrane receptor protein, Frizzled (Fz). Different combinations of Wnt and Fz ligand-receptor pairs can transduce at least three distinct kinds of intracellular signaling pathways. The "canonical" Wnt signaling pathway (Wnt/β-catenin pathway) results in changes of intracellular β-catenin levels and is thought to be involved in cell fate specification and proliferation. Wnt pathway activation can also lead to changes in either intracellular Ca^2+ ^concentration (Wnt/Ca^2+ ^pathway) or actin cytoskeleton reorganization (Wnt/tissue polarity pathway) [2]. The role(s) of these 'non-canonical' Wnt signaling pathway(s) in organ formation are largely unknown.

A function for Wnt signaling has been suggested by the expression patterns of *Wnt *and *Fz *genes during development of the mouse embryonic pancreas [3]. At embryonic day E11, *Wnt5a *and *Fz*-2 are expressed in the mesenchyme and epithelium of the pancreas. At E17.5, both *Wnt5a *and *Fz*-2 are co-localized with insulin- and glucagon-expressing cells. In contrast, only *Wnt5a *is expressed in the surrounding mesenchyme. *In situ *hybridization and RT-PCR gene expression analysis showed that both *Wnt5a *and *Fz*-2 are expressed in the embryonic pancreas from E11 until the end of gestation as well as postnatally. The highest level of expression is at E12 for *Wnt5a*, and at E12-E13 for *Fz*-2. Overexpression of *Wnt5a *in the pancreas results in the formation of multiple small and scattered islets, but the mechanism for such abnormality has not been characterized [3].

We explored a role of Wnt signaling in *insulin*-positive cell migration to form a pancreatic islet. In the mouse embryo at about E9.5, the primitive pancreatic epithelial cells express a transcription factor, *pdx*-1. *Glucagon*-positive cells are first detected around E10.5, and *insulin*-positive cells around E11.5 within the pancreatic ductal epithelium [4]. At E15.5, clusters of intermingled *insulin*-positive and *glucagon*-positive cells are found in the pancreatic interstitium, largely associated with the ducts [4,5]. During the last 4 days of gestation and postnatally, endocrine cells detach from the ducts, increase in number, and, at E17.5-E18.5, reorganize to form mature islets with the core of *insulin*-expressing cells surrounded by *glucagon*-expressing cells [6]. Formation of mature islets is thought to require migration of endocrine cells out of the pancreatic ductal epithelium to the pancreatic mesenchyme. These processes are partially controlled by matrix metalloproteinases (MMPs), a family of enzymes that degrade extracellular matrix proteins [7]. TGF-β signaling is necessary for the activation of MMP-2, which affects islet morphogenesis *in vitro *[8]. Recently, it has been reported that EGF signaling also regulates activation of MMP-2 and affects insulin-positive cell migration [6]. In mice lacking EGF-receptors, the majority of *insulin*-positive cells remain associated with pancreatic ducts in the newborn period.

The zebrafish pancreas functions similarly to that of other vertebrates by secreting hormones and exocrine enzymes to regulate blood glucose level and participate in digestion, respectively [9,10]. As in other vertebrates, synthesis and secretion of endocrine hormones in zebrafish occur in an islet called the Brockmann body, but unlike other vertebrates, only a single islet initially forms [11]. Thus, the zebrafish islet provides a model for the simplest endocrine pancreas with the core biological complexity of other vertebrates. *Insulin*-positive cells are specified as bilateral patches around the 14-somite stage and subsequently form a single islet in the midline [12]. Immunohistochemical studies using antibodies against insulin and glucagon revealed that the zebrafish islet consists of a core of *insulin*-expressing cells surrounded by *glucagon*-expressing cells, a structural organization similar to that observed in the mouse and the human [11,12]. However, the pattern of cell migration and the molecular mechanisms of zebrafish islet morphogenesis have not been previously investigated.

We characterized the process of *insulin*-positive cell migration in zebrafish and examined a role of Wnt signaling in pancreatic islet formation in zebrafish and the mouse. We show that *wnt-5/fz-2 *signaling is required for proper islet formation in zebrafish, and we demonstrate that *Wnt5a *signaling is required for the separation of islets from the ducts in the mouse. These phenotypes in zebrafish and mouse are consistent with defects in *insulin*-positive cell migration demonstrating a new and conserved role of Wnt signaling in vertebrate endocrine pancreas formation.

## Results

### Loss of direction results in scattered *insulin*-positive cells in embryos injected with *fz*-2 morpholinos

**Figure 1:**
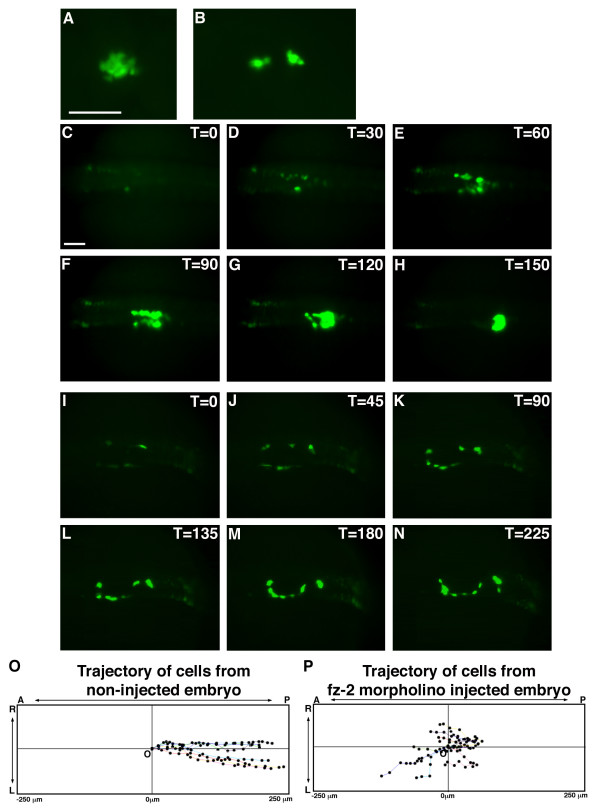
**Time-lapse imaging of insulin:GFP transgenic embryos shows cell migration defects in *Fz-2 *morphants**. (A, C-H) Uninjected insulin:GFP transgenic embryo, (B, I-N) fz-2 MO-injected insulin:GFP transgenic embryo. All panels are dorsal views and anterior is to the left. Scale bar represents 100 μm. (A) Uninjected transgenic embryo, 24 hpf. (B) *Fz*-2 MO-injected transgenic embryo, 24 hpf. (C) At the 14-somite stage, bilateral patches of GFP-positive cells are visible in uninjected embryo. (D) At the 15–16 somite stage, GFP-positive cells have started proliferating. (E-G) At the 17 somite to 24 hpf stages, GFP-positive cells are aligned in bilateral rows of cells and undergo a medial and posterior migration. (H) At 24 hpf, all GFP-positive cells have merged to form one islet. (I) At the 14-somite stage, bilateral patches of GFP expression are apparent in *fz*-2 MO-injected embryos similar to uninjected embryos. (J-M) GFP-positive cells migrate in random directions in fz-2 morphant embryos. (N) At 24 hpf, GFP-positive cells have still not merged. (O) Trajectory of GFP-positive cells in uninjected insulin:GFP embryo. Notice that cells are uniformly moving posteriorly. (P) Trajectory of GFP-positive cells in *fz*-2 MO-injected insulin:GFP embryo. Notice cells are moving in random directions. A: anterior, P: posterior, T: time, L: left, R: right, O: origin.

To study the function of *fz-2*, we used morpholino-modified oligonucleotides (MOs) as sequence-specific translational inhibitors in zebrafish [13]. Injection of *fz*-2 MOs [14] into transgenic zebrafish embryos ('morphants') carrying the GFP reporter gene under the control of the *insulin *promotor (insulin:GFP transgenic fish: [15]) resulted in scattered GFP-positive cells compared to the single islet observed in wild-type (WT) embryos (Fig. 1A,B). To determine the cause of abnormal *insulin *expression in FZ-2 morphants, we conducted a time-lapse imaging analysis. In wild-type embryos, bilateral *insulin*-positive cells first appeared at approximately the 14-somite stage. These cells divided and actively migrated to the posterior. As insulin-positive cells migrate, these cells converge in the midline. By the 24 hours-post-fertilization (hpf) stage, all *insulin*-positive cells associate to form a single islet at the midline (Fig. 1C–H [also see additional file 1]). We have also performed a time-lapse analysis on insulin:GFP transgenic embryos injected with rhodamine-conjugated dextran (Molecular Probe, D-1841). This created embryos with GFP expression in the insulin-positive cells and rhodamine localized to random cells in a mosaic pattern. We found that the GFP-negative cells labeled with rhodamine did not change their relative positions and did not show any morphological changes associated with migrating cells, while GFP-positive cells moved posteriorly and medially, arguing that the GFP positive cells move relative to their neighboring cells [see additional file 2]. Plotting the trajectory of GFP-positive cells demonstrated that the pattern of cell migration is mostly along a straight line from the initial to the final position (Fig. 1O). The average migration rate of a GFP positive cell was 0.3 ± 0.12 μm/minute and average A-P progression was 0.3 ± 0.04 μm/minute (n = 10).

In transgenic insulin:GFP zebrafish embryos injected with *fz*-2 MO, bilateral *insulin*-positive cells also appeared at the 14-somite stage, as observed in uninjected transgenic embryos. However, these cells failed to migrate to the posterior. At 24 hpf, *insulin*-positive cells remained scattered in *fz-2 *morphants (Fig. 1I–N [also see additional file 3]). The average migration rate of a GFP-positive cell was 0.3 ± 0.08 μm/minute, similar to uninjected transgenic embryos, indicating that the GFP-positive cells are capable of moving at the normal speed. However, the trajectory of *insulin*-positive cells in *fz*-2 MO-injected embryos showed that the cells were moving randomly (Fig. 1P). The average A-P progression of an *insulin*-expressing cell in *fz-2 *morphant embryos was -0.02 ± 0.07 μm/minute (n = 10). These data argue that GFP-positive cells still can move normally but have lost directional information in *fz-2 *MO-injected transgenic embryos.

### *Fz-2 *and *insulin *are expressed in neighboring cells

**Figure 2:**
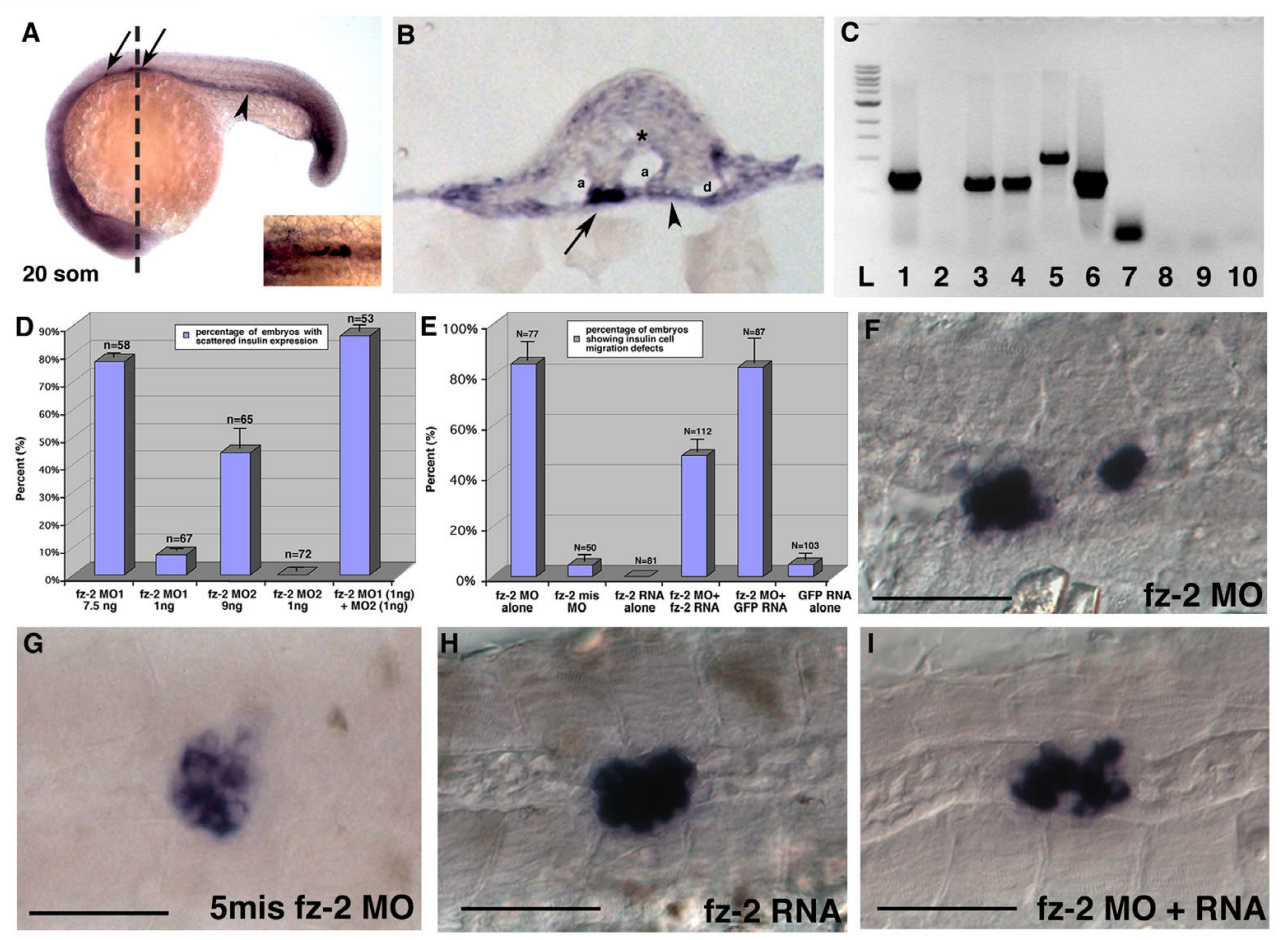
**Migration defects in *Fz-2 *morphant embryos can be rescued by synthetic *fz-2 *mRNA**. (A) Double *in situ *hybridization with *fz*-2 and *insulin *at 20 somite stage of development. Arrow, *insulin*; arrowhead, *fz-2 *expression in the endoderm; dotted line, approximate position of the section in (B). (B) A section of double in situ hybridization with *fz-2 *and *insulin*. *Fz-2 *is expressed more strongly on the surface of mesoderm and entire endoderm. Arrow, *insulin*; arrowhead, *fz-2 *expression in the endoderm; a, arteries; asterisk, neural tube; d, pronephric duct. (C) RT-PCR using cDNA made from sorted cells of transgenic insulin:GFP zebrafish embryos. L: ladder; lanes 1–5: GFP-negative cells; lanes 6–10: GFP-positive cells; lanes 1, 6: EF1α lanes2, 7: *insulin*; lanes 3, 8: *fz-2 *primer set #1; lanes 4, 9: *fz-2 *primer set #2; lanes 5, 10: *wnt-5*. (D) High-dose injection of either *fz-2 *MO1 or MO2 resulted in scattered *insulin *expression, whereas low dose injection of either MO caused such defects in less than 10% of embryos. Co-injection of low dose *fz-2 *MO1 and MO2 resulted in synergistic increase of percentage of embryos with scattered *insulin *expression. (E) 80% of *fz*-2 MO-injected embryos displayed scattered *insulin *expression. Co-injection of *fz*-2 MO and *fz*-2 RNA reduced the percentage of embryos with abnormal *insulin *expression down to 45%. (F-I) *In situ *hybridization with *insulin *at 24 hpf stage, anterior is to the left, (F) *fz*-2 MO1-injected embryo, (G) *fz-2 *mismatch MO-injected embryo, (H) *fz*-2 RNA-injected embryo, (I) *fz*-2 MO- and *fz*-2 RNA-co-injected embryo. Notice the compact islet in this embryo that displays an undulated notochord.

As we reported earlier, *fz-2 *is restricted to somitic mesoderm and posterior paraxial mesoderm starting at the one somite stage. This expression pattern remains similar during somitogenesis, although the expression level of fz-2 in somitic mesoderm gradually decreases after 12–14 somite stage. (Fig. 2A, [14]). Double *in situ *hybridization of *insulin *and *fz-2 *and subsequent sectioning of the embryo showed that *fz-2 *expression is concentrated on the surface of somatic mesoderm and the entire endoderm adjacent to *insulin*-expressing cells (Fig. 2A,B). To determine if *fz-2 *is expressed in the *insulin*-positive cells, we sorted GFP-positive and -negative cells from the 20 somite stage insulin:GFP trangenic zebrafish embryos using Fluorescent Automated Cell Sorting (FACS), isolated total RNA from each sample, synthesized cDNA, and performed RT-PCR analysis for the presence of *fz-2 *transcript in the GFP-positive cells. As expected, EF-1α, a positive control, but not *insulin *was detected in the GFP-negative cells (Fig. 2C). In contrast, both EF1-α and *insulin *transcripts were detected in GFP-positive sample (Fig. 2C) indicating that the cell sorting procedure was effective. Two different sets of primers designed to amplify *fz-2 *transcripts produced bands from the GFP-negative cells but not from the GFP-positive cells (Fig. 2C). Our analysis shows that even though *fz-2 *and *insulin *are expressed in neighboring cells as detected by *in situ *hybridization, they are not co-expressed in the same cells.

### *Fz-2 *plays a specific role in pancreatic islet formation

To confirm the identity of the scattered GFP-positive cells in *fz*-2 MO-injected insulin:GFP embryos, *fz-2 *morphant embryos were fixed at 24 hpf and analyzed for *insulin *expression by *in situ *hybridization. At 24 hpf, wild-type zebrafish embryos have a single islet consisting of 15–20 cells at the midline. In contrast, *insulin*-expressing cells in *fz-2 *morphant embryos were scattered along the A-P axis similar to the pattern of GFP-expressing cells in *fz*-2 MO-injected transgenic embryos (Fig. 2F). The number of *insulin*-positive cells in *fz-2 *morphants was not significantly different from control embryos despite the abnormal islet morphology, indicating that cell proliferation is not significantly affected (average: 12 ± 2 cells in wild-type and 10 ± 2 cells in MO-injected, n = 25 for each group).

Injection of two different previously published MOs targeting non-overlapping 5' regions of *fz-*2 mRNA generated similar effects on insulin-expressing cell migration. Furthermore, co-injection of two *fz*-2 MOs resulted in a synergistic increase in a percentage of embryos with scattered insulin expression, suggesting a specific role of *fz-*2 in proper islet formation (Fig. 2D). To assess whether scattered *insulin *expression observed in *fz*-2 MO-injected embryos was specific to the loss of *fz-2 *function, we also injected a five-base mismatch *fz*-2 MO, which elicited no effect on *insulin*-expressing cell migration (Fig 2E,G). To test the specificity of the observed phenotype in *fz-*2 MO-injected embryos further, we examined the ability of synthetic *fz*-2 mRNA to reverse the observed defects in *insulin*-positive cell migration. The injected synthetic *fz*-2 mRNA contains a β-globin 5' leader sequence and does not contain the *fz-*2 MO target-site sequence. Injection of *fz*-2 MO resulted in 84% of the embryos (n = 77) with scattered *insulin *expression (Fig. 2E,F). None of the *fz*-2 mRNA-injected embryos (n = 81) displayed scattered *insulin *expression (Fig. 2E,H). In contrast, co-injection of *fz*-2 MO and *fz*-2 mRNA resulted in a significantly lower frequency of embryos (n = 112, p value = 0.001) with scattered *insulin *expression compared with embryos injected with *fz*-2 MO only (Fig. 2E,I). These results demonstrate that the scattered *insulin *expression observed in *fz-2 *morphant embryos is specific to the loss of *fz*-2 function.

### *Wnt-5 *has a similar and specific role in islet formation

**Figure 3:**
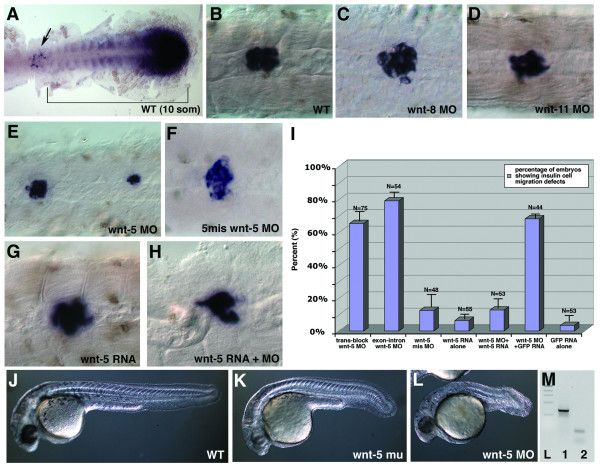
***Wnt*-5 has a specific role in islet formation**. (A) Double *in situ *hybridization with *pdx*-1 and *wnt*-5, 10 som stage, dorsal view, the anterior is to the left. Arrow, *pdx*-1 expression, bracket, *wnt-5 *expression. (B-H) *In situ *hybridization with insulin at 24 hpf. (B) wild-type, (C) WNT-8 morphant embryos, (D) WNT-11 morphant embryos, (E) WNT-5 morphant embryos, (F)*wnt*-5 mismatch MO-injected embryos, (G) *wnt*-5 RNA injected embryo, (H) *wnt*-5 MO and *wnt*-5 RNA co-injected embryo. Notice the compact islet in this embryo that displays an undulated notochord. (I) Percentage of embryos with scattered *insulin *expression resulting from injection of *wnt*-5 MO reduced significantly from 60% to 10% when *wnt*-5 RNA was co-injected with *wnt*-5 MO. (J-L) Morphology at 24 hpf, (J) wild-type, (K) *wnt-5 *insertional mutant, (L) *wnt-5 *translation-blocking MO-injected embryos. Notice that *wnt-5 *MO injected embryos have more severe morphological phenotype than *wnt-5 *insertional mutant embryos. (M) RT-PCR analysis of *wnt-5 *transcript in *wnt-5 *exon-intron MO injected embryos. Injection of *wnt-5 *exon-intron MO results in severely shortened *wnt-5 *transcript. L:ladder, 1:EF-1α control, 2:*wnt-5*.

Because the Fz protein family can function as receptors of Wnt proteins, we wanted to test if any known Wnt protein is similarly required for *insulin*-positive cell migration. Loss-of-function of three Wnts – *wnt*-5, -8, and -11 – are each reported to cause developmental abnormalities grossly similar to the ones observed in *fz-2 *morphants [16,17]. Among these, *wnt*-5 exhibits the most similar expression pattern to *fz*-2 during somitogenesis. Zebrafish *wnt*-5 is weakly expressed in the posterior half of the somitic and lateral mesoderm and strongly in the tail bud during somitogenesis [18]. Interestingly, double *in situ *hybridization against *wnt*-5 and *pdx*-1 revealed that *wnt*-5 is expressed as a gradient of RNA from the posterior limit of *pdx*-1 expression to the tail-bud at the 10-somite stage when *pdx*-1 is first detected (Fig. 3A). However, we did not detect wnt-5 in the GFP-positive cells isolated from insulin:GFP transgenic zebrafish embryos indicating that *wnt-5 *is not co-expressed with *insulin *(Fig. 2C, lane 10: compare to wnt-5 band from GFP-negative cells in lane 5).

To identify a candidate Wnt ligand, we utilized injections of MOs against *wnt*-5, -8, and -11 that effectively generate mutant phenocopies [17,19]. We generated Wnt-5, -8, and -11 morphants as described and analyzed *insulin *expression by *in situ *hybridization at 24 hpf [17,19]. Islet formation was normal in Wnt-8 and -11 morphant embryos as analyzed for the expression of insulin, pdx-1, glucagon and somatostatin (Fig. 3B–D, data not shown). In contrast, analysis of *insulin *expression at 24 hpf of Wnt-5 morphant embryos revealed scattered *insulin *expression along the A-P axis, similar to the islet phenotype noted in *fz-2 *morphant embryos (Fig. 3E).

To address the specificity of *wnt-5 *function in pancreatic islet formation, we designed a second MO targeting the 3' splice-site of exon 3. Injection of this MO resulted in effective skipping of exons 2 and 3, leading to a transcript that is only about 200 bp in length (Fig. 3M). When we injected this MO, embryos showed scattered *insulin *expression similar to embryos injected with *wnt-5 *translation blocking MO (Fig. 3I), arguing that the zygotic function of *wnt-5 *is involved in *insulin*-positive cell migration. In addition, injection of a five-base-mismatch *wnt*-5 MO resulted in embryos with normal *insulin *expression (Fig. 3F,I). Furthermore, the observed scattered *insulin *expression pattern in Wnt-5 morphant embryos was ameliorated by the addition of *wnt*-5* mRNA that encodes an altered *wnt5 *open reading frame engineered with degenerate nucleotides in the region around the translation start site to avoid targeting by the *wnt*-5 MO (see Methods). When translation blocking *wnt*-5 MO was injected alone, 65% of embryos (n = 75) showed scattered *insulin *expression (Fig. 3E,I). Embryos injected with *wnt*-5* mRNA alone resulted in 94% of embryos (n = 55) with normal *insulin *expression (Fig. 3G,I). The percentage of embryos with abnormal *insulin *expression decreased to 12% (n = 53, p value = 0.003) when embryos were co-injected with *wnt*-5* mRNA (Fig. 3H,I).

We also analyzed *insulin *expression in two different published *wnt-5 *mutant alleles (alleles of the mutant locus *pipetail*; kind gifts of Dr. M. Hammerschmidt (*ppt*^*ti*265^) [20] and Dr. N. Hopkins(*ppt*^*hi*1780*b*^) [21]). *In situ *analysis using the *insulin *marker did not detect any significant effect on pancreas development in embryos homozygous for either of the tested *pipetail *alleles (data not shown). Gross morphological examination of embryos from either *pipetail *allele demonstrated a significant inter-embryo variation in manifestation of the *pipetail *embryonic phenotype, with the insertional allele (*ppt*^*hi*1780*b*^) exhibiting an overall more severe phenotype. The *ppt*^*hi*1780*b *^allele showed phenotypes that were less extreme than the effects noted for the *wnt-5 *morphants (Fig. 3J–L). To investigate further whether the scattered *insulin *expression observed in WNT-5 morphant embryos is specific to the loss of WNT-5 function, we tested to see if carriers of the *ppt*^*hi*1780*b *^allele are more sensitive to *wnt-5 *MO injection (Table 1). Injecting 2 ng of either translation-blocking or exon-intron junction targeting *wnt-5 *morpholinos in wild-type embryos resulted in less than 5% of embryos with scattered *insulin *expression. We injected the same low dose *wnt-5 *MOs into embryos obtained from a cross between *wnt-5 ppt*^*hi*1780*b *^allele carrier and wild-type adult fish. In this batch of embryos, approximately 50% are expected to be carriers of the *ppt*^*hi*1780*b *^allele. This study resulted in approximately 50% of embryos exhibiting scattered *insulin *expression (Table 1). Furthermore, genotyping of individual embryo showed that 90% of embryos with abnormal insulin expression were *ppt*^*hi*1780*b *^carriers (n = 20) whereas 100% with normal insulin expression were wild-type (n = 20), arguing that carriers of the *ppt*^*hi*1780*b *^allele are more sensitive to *wnt-5 *MO injection [see additional file 4]. These data argue that the observed *wnt*-5 MO phenotype is specific to the targeting of the *wnt-5 *gene and that zygotic *wnt-5 *function is required for normal pancreatic islet formation.

**Table 1:** *Wnt-5 *heterozygous embryos are more sensitive to *wnt-5 *MO injection in pancreatic islet formation.

	WT		WT X *wnt*-5/WT	
	trans-block wnt-5 MO	exon-intron wnt-5 MO	trans-block wnt-5 MO	exon-intron wnt-5 MO

% of embryos with abnormal insulin expression	5 ± 2%	0%	46 ± 5%	52 ± 3%
total # of embryos	N = 124	N = 113	N = 53	N = 57

2 ng of either *wnt-5 *translation-blocking or exon-intron junction targeting morpholinos were injected into either wild-type embryos or embryos generated from an outcross of a *wnt-5 *mutant carrier adult. In wild-type embryos, injection of either *wnt-5 *MO resulted in less than 5% of embryos with scattered *insulin *expression. However, injection of either *wnt-5 *MO resulted in about 50% of embryos with scattered *insulin *expression in embryos obtained from the *wnt-5 *mutant carrier outcross.

### WNT-5 and FZ-2 morphant embryos display a similar pancreatic developmental defect

**Figure 4:**
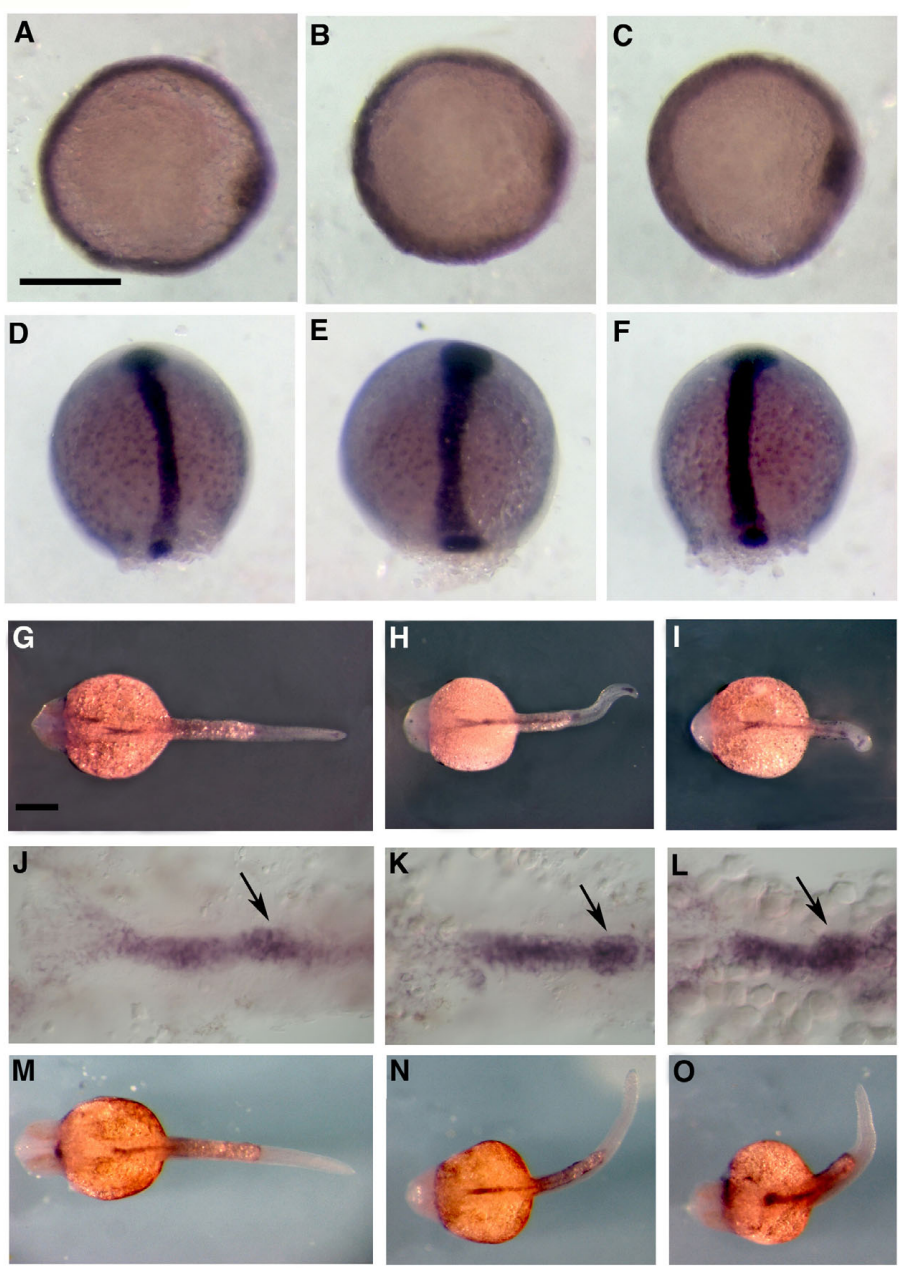
**Early endoderm markers are not affected in *Wnt-5 *and *Fz-2 *morphant embryos**. All pictures are dorsal views. (A, D, G, J, M) wild-type, (B, E, H, K, N) *Fz-2 *morphants, (C, F, I, L, O) *Wnt-5 *morphants. (A-C) *mixer*, 50% epiboly, (D-F) *sox*-17, 90% epiboly, (G-I) *fox-A3*, 24 hpf, (J-L) anterior endoderm expression of *fox-A3*, arrow, pancreatic endoderm, 24 hpf, (M-O) *gata*-6, 24 hpf. Scale bar = 300 μm.

To determine which step of pancreatic development is perturbed, we analyzed the expression patterns of different markers in *fz-2 *and *wnt-5 *morphant embryos. We examined early endodermal markers, *mixer *at 50% epiboly stage and *sox*-17 at 90% epiboly stage in *fz-2 *and *wnt-5 *morphant embryos, and found that the expression of *mixer *and *sox*-17 were normal in those embryos (Fig. 4A–C, 4D–F). We also examined the anterior endoderm marker, *fox-A2*, and the posterior endoderm marker, *gata*-6, at 24 hpf in wild-type, *fz-2 *and *wnt-5 *morphant embryos. Expression of *fox-A2 *and *gata*-6 was normal in both *fz-2 *and *wnt-5 *morphant embryos (Fig. 4G–I, 4J–L). These results indicate that the abnormal *insulin *expression observed in both *fz-2 *and *wnt-5 *morphant embryos is not due to secondary defects because of abnormal endoderm specification or migration.

**Figure 5:**
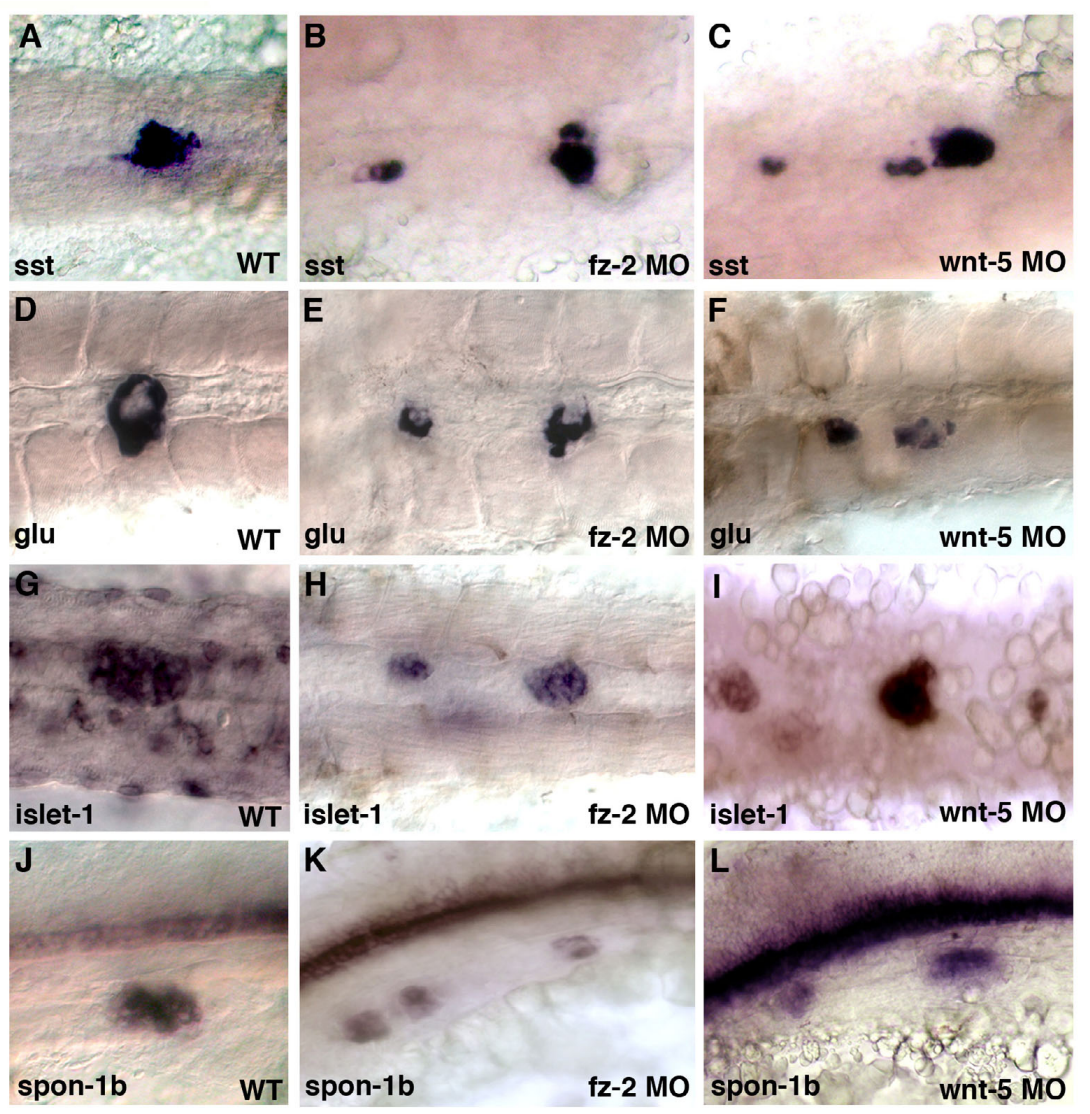
***Wnt-5 *and *Fz-2 *morphant embryos exhibit similar pancreatic islet defects at 24 hpf**. In all panels, anterior is to the left and 24 hpf. .A-I, dorsal view; J-L, lateral view. (A, D, G, J) Wild-type embryos. (B, E, H, K) Fz-2 morphants. (C, F, I, L) Wnt-5 morphants. *In situ *hybridization analysis of (A, B, C) *somatostatin*, (D, E, F) *glucagon*, notice a hollow spot in the middle of each patch, (G, H, I) islet-1, (J, K, L) *fspondin-2b*. Note scattered pancreatic cells in Fz-2 and Wnt-5 morphants.

At 24 hpf, *somatostatin*, a marker for mature pancreatic δ-cells, was scattered similarly to *insulin *in both *fz-2 *and *wnt-5 *morphant embryos (Fig. 5A–C). *Glucagon*, which is expressed in pancreatic endocrine α-cells, was scattered along the A-P axis but still expressed at the edge of the islet in both *fz-2 *and *wnt-5 *morphants (Fig. 5D–F). Other endocrine pancreas markers, *islet*-1 and *fspondin*-*2b*, were also scattered along the A-P axis (Fig. 5G–I, 5J–L).

**Figure 6:**
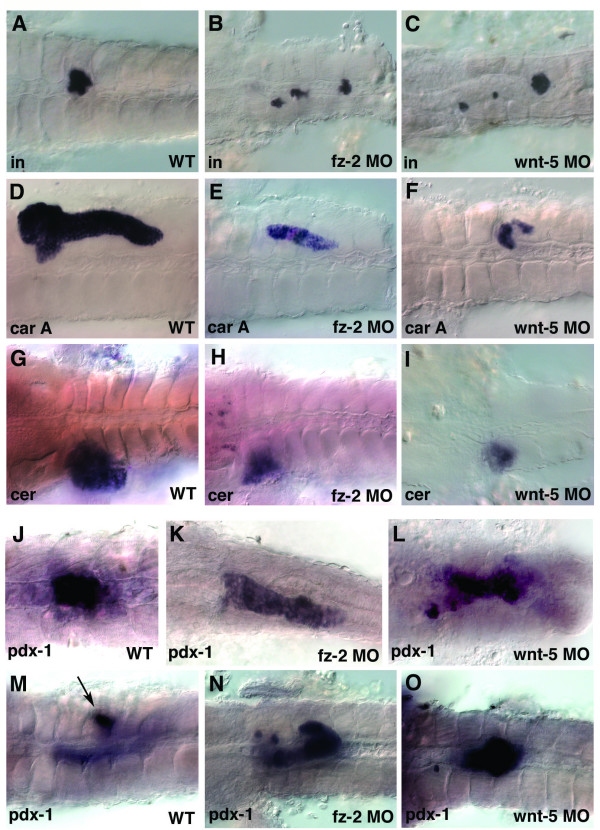
***Wnt-5 *and *Fz-2 *morphant embryos have other similar defects**. In all panels, view is dorsal, anterior is to the left. (A-I, M-O) 3dpf, (J-L) 24 hpf stage. (A, D, G, J, M) Wild-type embryos. (B, E, H, K, N) *Fz-2 *morphants. (C, F, I, L, O) *Wnt-5 *morphants. *In situ *hybridization analysis of (A-C) *insulin*, (D-F) *carboxypeptidase A*, notice the hollow spot indicating the position of the islet, (G-I) *ceruloplasmin*, (J-O) *pdx-1*, (M) arrow, *pdx-1*-staining in islet.

At the 3dpf stage, *insulin*-positive cells remain scattered in both *wnt-5 *and *fz-2 *morphants (Fig. 6A–C). Expression of an exocrine pancreas marker, *carboxypeptidase-A*, and a liver marker, *ceruloplasmin*, was reduced in both *fz-2 *and *wnt-5 *morphant embryos (Fig. 5D–I respectively). The hollow spot indicating the position of the islet within the exocrine pancreas was not observed in either *wnt-5 *or *fz-2 *morphants (Fig. 5D–F). This indicates that pancreatic islets in *wnt-5 *and *fz-2 *morphants are not completely embedded within exocrine tissue as found in wild-type embryos. However, scattered *insulin *expression is not likely to be affected by the reduction of the exocrine pancreas, because the exocrine pancreas in zebrafish does not develop until after the completion of *insulin*-positive cell migration.

Our results suggest that *fz*-2 and *wnt*-5 have similar function in pancreatic islet development. Interestingly, examination of the transcription factor *pdx*-1 expression profile revealed an unexpected phenotype in these embryos. *Pdx*-1 is one of the earliest known markers for the entire pancreas and is important for both early pancreatic development and adult β-cell function [22]. The area of *pdx*-1 expression was expanded in both *fz-2 *and *wnt-5 *morphants as analyzed by *in situ *hybridization at 24 hpf (Fig. 6J–L). At 3dpf, *pdx*-1 expression is restricted to the pancreatic islet, duct, and part of the intestine (Fig. 6M). *Pdx-1*-expressing cells in *fz-2 *and *wnt-5 *morphant embryos remain scattered and do not coalesce into a single islet until 3dpf (Fig. 6K,L) similar to *insulin*-expressing cells. In addition, the area of *pdx-1 *expression in the intestine becomes reduced in *fz-2 *and *wnt-5 *morphant embryos compared to wild-type embryos at 3dpf (Fig. 6M–O). The expanded *pdx*-1 expression at 24 hpf and misexpression at 3dpf in *fz-2 *and *wnt-5 *morphants requires further study (see discussion).

### *Wnt-5 *and *fz-2 *can genetically interact

**Figure 7:**
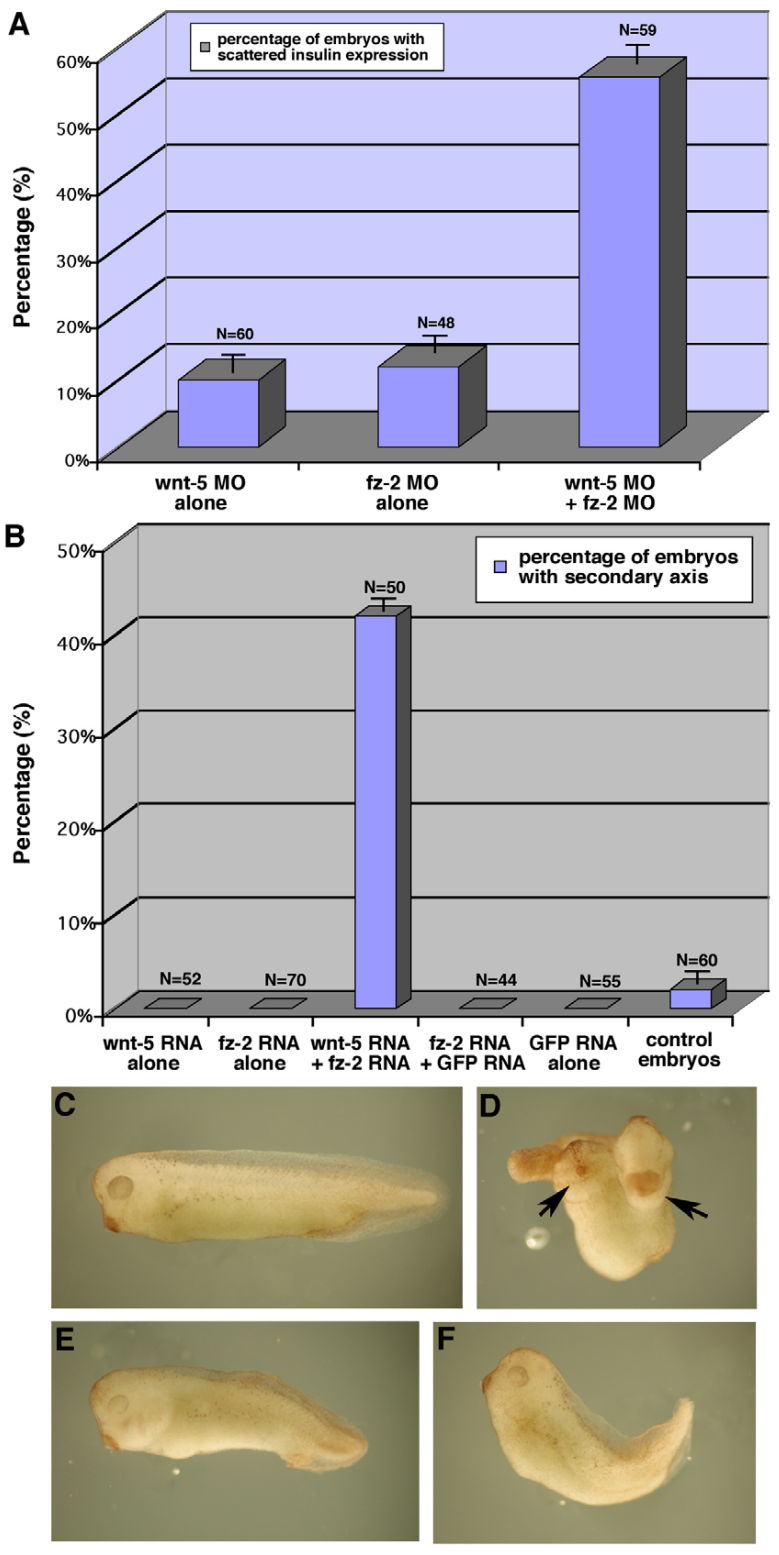
***Wnt*-5 and *fz*-2 are in the same signaling pathway**. (A) Injection of either *wnt*-5 MO or *fz*-2 MO mix results in less than 10% of embryos with scattered insulin expression. Co-injection of *wnt*-5 and *fz*-2 MOs results in 50% of embryos with defects. (B) Injection of either *wnt*-5 mRNA or *fz*-2 mRNA did not cause secondary axis in *Xenopus *embryos, whereas co-injection with both mRNAs resulted in 40% of embryos with secondary axis. Control injections of GFP mRNA alone or together with *fz*-2 mRNA resulted in no embryos with secondary axis. (C-F) *Xenopus *embryos, tailbud stage, (C) wild-type, (D) *wnt*-5 and *fz*-2 mRNA co-injected, black arrows-point to the primary and secondary hatching glands, (E) *wnt*-5 mRNA injected, (F) *fz*-2 mRNA injected.

We next determined if *wnt*-5 and *fz*-2 could interact genetically. First, we co-injected low doses of *wnt*-5 and *fz*-2 MOs. If *wnt*-5 and *fz*-2 were in two independent signaling pathways, co-injection of MOs against these genes would result in an additive increase of embryos with abnormal *insulin *expression. In contrast, a synergistic increase of embryos with abnormal *insulin *expression would indicate that *wnt-5 *and *fz-2 *are either in the same signaling pathway or in two pathways that can interact in the normal *insulin*-positive cell migration process. Injection of a low dose of *wnt-*5 MO resulted in 10% of embryos (n = 60) with abnormal *insulin *expression. Injection of a low dose of *fz*-2 MOs resulted in 12% of embryos (n = 48) with abnormal *insulin *expression. When we co-injected *wnt*-5 MO and *fz*-2 MOs, 55% of embryos (n = 59) displayed abnormal *insulin *expression, a synergistic increase in the percentage of embryos with abnormal *insulin *expression (Fig. 7A). This result suggests that *wnt*-5 and *fz*-2 can interact genetically in the same signaling pathway during islet formation.

To test a possible direct interaction between zebrafish *wnt*-5 and *fz*-2 further, we used a *Xenopus *secondary axis induction assay [23]. In this system, a Fz-dependent induction of the secondary axis in *Xenopus *has been shown to be the result of direct receptor (Fz) activation by the Wnt ligand. This effect can be mediated by Wnt ligands and Fz receptors that normally function via either canonical or non-canonical signaling processes. In this experiment, we injected either *wnt*-5 mRNA or *fz-2 *mRNA into two ventral blastomeres of 4-cell stage *Xenopus *embryos. Injecting zebrafish *wnt-5 *RNA or *fz*-2 RNA elicited a shortened and mildly bent body at the tailbud stage without the induction of any secondary axes (Fig. 7B,E,F). When we co-injected both RNAs in *Xenopus *embryos, more than 50% of embryos showed a secondary axis at the tailbud stage (Fig. 7B,D). This suggests that zebrafish Wnt-5 and Fz-2 proteins can interact functionally and signal when placed in an appropriate cellular environment.

### *Fz*-2 is epistatic to *wnt*-5

**Figure 8:**
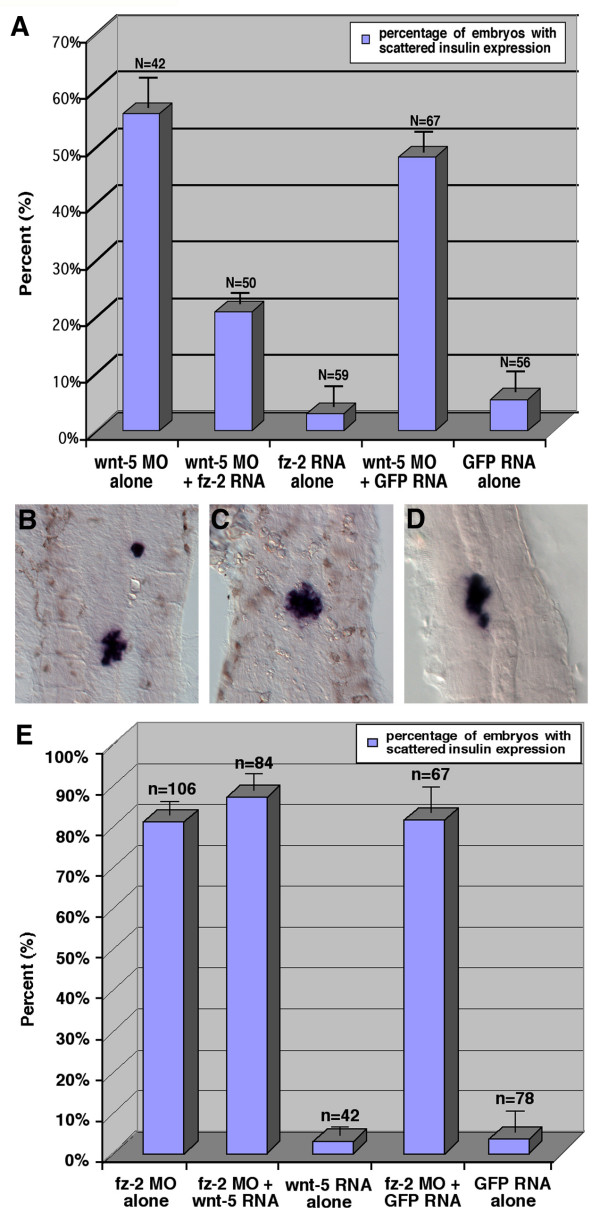
***Wnt*-5 acts genetically upstream of *fz*-2**. (A) Injection of *wnt*-5 MO alone results in 50% embryos with insulin cell defect. Injection of *fz*-2 mRNA results in 5% embryos with insulin cell defects. Co-injection of *wnt*-5 MO and *fz*-2 mRNA results in 20% of embryos with abnormal insulin expression. In a control experiment, co-injection of *wnt*-5 MO and GFP mRNA results in 45% of embryos with defects. (B-D) *Insulin *expression as analyzed by *in situ *hybridization at 24 hpf, (B) *wnt*-5 MO injected, (C) *fz*-2 RNA injected, (D) *wnt*-5 MO- and *fz*-2 RNA-injected embryos. Note that *fz-2 *mRNA rescues *insulin *cell migration defect in *wnt-5 *morphants. (E) Same dose of *wnt-5 *mRNA that can rescue the *insulin *cell migration defects in *wnt-5 *morphants cannot rescue the defects in *fz-2 *morphants.

To determine if *wnt*-5 acts genetically upstream of *fz*-2, we removed Wnt-5 protein by injecting *wnt*-5 MO and assayed whether *fz-2 *mRNA can rescue the resulting migration defects. When *wnt*-5 MO was injected alone, 56% of injected embryos (n = 42) showed scattered *insulin *expression (Fig. 8A,B). Injection of *fz*-2 RNA alone did not cause significant abnormalities in *insulin *expression (3% of embryos, Fig. 8A,C). The percentage of embryos with scattered *insulin *expression significantly decreased to 20% (n = 50, p value = 0.019) when *fz*-*2 *RNA was co-injected with *wnt*-5 MO (Fig. 8A,D). In a control experiment, co-injection of *wnt*-5 MO and GFP RNA did not reduce the percentage of embryos with abnormal *insulin *expression (Fig. 8A).

In contrast, injection of *wnt*-5 RNA cannot ameliorate defects in *fz-2 *morphant embryos (Fig. 8E). Injecting *fz*-2 MO resulted in 80% of embryos (n = 106) with scattered *insulin *expression. Injecting *wnt*-5 RNA caused 5% of embryos (n = 42) with scattered *insulin *expression. Co-injection of *fz*-2 MO and *wnt*-5 RNA resulted in 88% of embryos (n = 84) with scattered *insulin *expression. These results demonstrate that *fz-2 *RNA can rescue abnormal islet morphology caused by the *wnt*-5 MO, whereas *wnt-5 *RNA cannot rescue abnormal islet morphology caused by *fz*-2 MO, placing *fz*-2 genetically downstream of *wnt*-5 in this process.

### *Wnt-5a *knockout mice exhibit similar islet precursor cell migration defects and abnormal islet morphology

**Figure 9:**
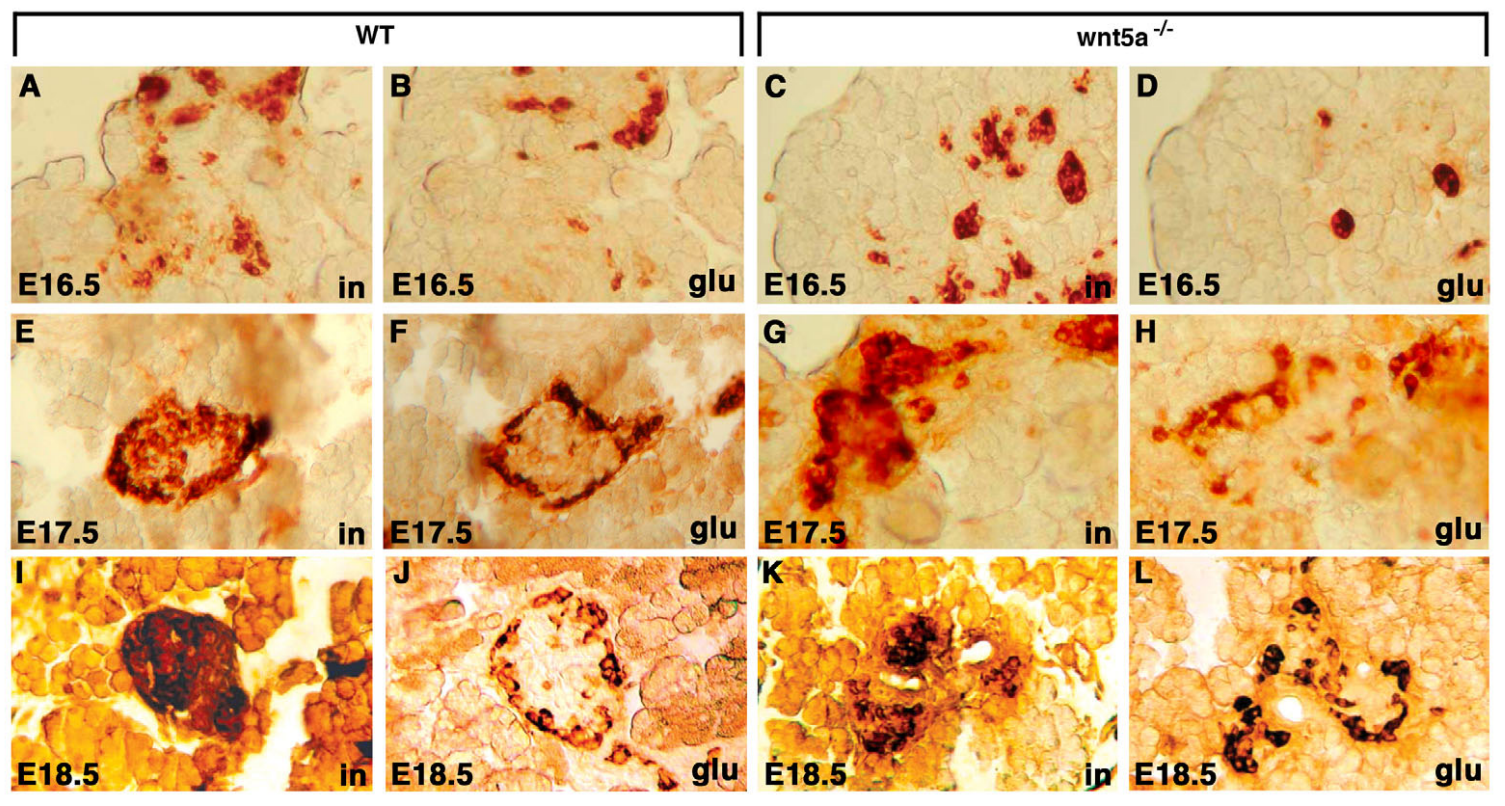
**Pancreatic islet development in *Wnt5a*^-/- ^mouse embryos is not delayed**. (A-D) E16.5, (E-H) E17.5, (I-L) E18.5, (A, C, E, G, I, K) insulin antibody staining, (B, D, F, H, J, L) glucagon antibody staining, (A, B, E, F, I, J) pancreas tissue from wild-type siblings, (C, D, G, H, K, L) pancreas tissue from *Wnt5a*^-/- ^mouse embryos. Notice that glucagon staining is round and spherical at E16.5, but positioned at the periphery of insulin cells at E17.5 and E8.5.

To test if the role of Wnt-5 signaling in pancreatic development is conserved between different vertebrates, we obtained mice heterozygous for a previously described *Wnt5a *null allele [24]. Wild type and *Wnt5a*^-/- ^embryos were harvested at E16.5, E17.5 and E18.5 and examined for defects in pancreatic development. Although *Wnt5a*^-/- ^embryos have defects in many structures, the size and macroscopic morphology of the pancreas were normal with a characteristic loose lobular appearance and normal anatomical positioning: with the head of the pancreas located at the curve of the duodenum, the body associated with the greater omentum at the ventro-posterior surface of the stomach, and the narrower tail region pointed towards the hilum of the spleen (data not shown). We next performed immunohistochemical analysis of consecutive pancreatic sections with antibodies against insulin and glucagon between E16.5, E 17.5 and E18.5. In both wild type and mutant pancreata at E16.5, insulin-positive cells were intermingled with glucagon-positive cells as described previously [4] and did not have a typical mature distribution with insulin cells located centrally and glucagon cells located peripherally (Fig. 9A–D). At E17.5, insulin-staining cells were becoming more compact and glucagon-staining cells began to assume a peripheral distribution in both wild type and mutant mice (Fig. 9E–H). Since this distinct cell architecture is not observed in wild type mice before E17.5 [4,25], the appearance of the peripheral distribution of glucagon-positive cells in the mutant islets at E17.5 argues against maturational delay.

**Figure 10:**
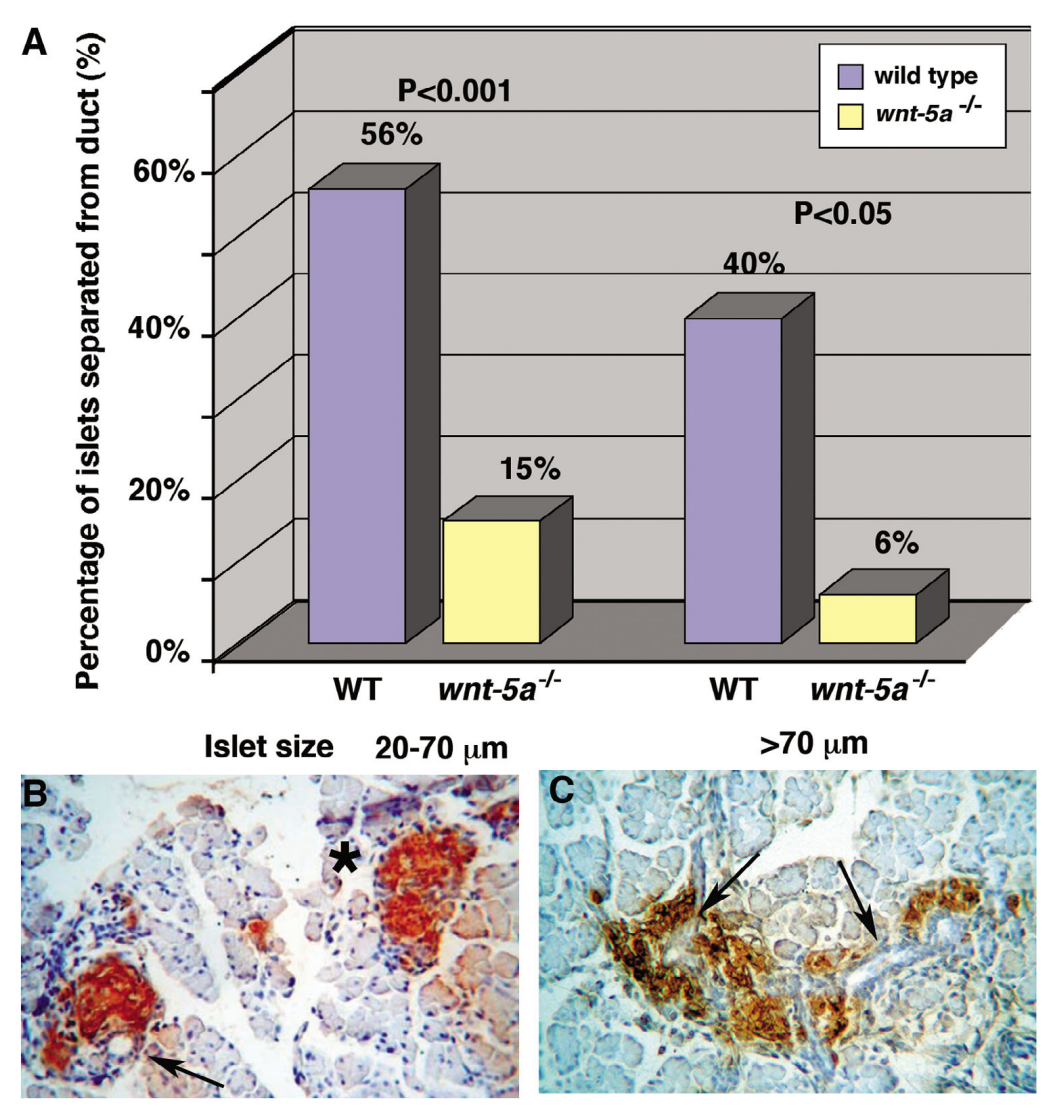
***Wnt5a*^-/- ^islets remain in ductal proximity and have a streaked appearance at E18.5**. (A) In *Wnt5a*^-/- ^embryos, most islets are associated with ducts. Both small and large β-cell aggregates are more frequently associated with pancreatic ducts in *Wnt5a*^-/- ^embryos at E18.5 than in wild-type embryos. (B, C) Insulin antibody staining. (B) Round and compact islets in wild-type embryos. A normal pancreas consists of islets that are associated and separated from ducts. Arrows: pancreatic duct, asterisk: an islet separated from duct. (C) Streak-like, fragmented islets in *Wnt5a*^-/- ^mutant embryos.

Similar pattern was observed in the islets at E18.5 with insulin-producing β-cells in the center of the islet (Fig. 9I,K) and the glucagon-producing α-cells on the periphery of the islet (Fig. 9J,L). However, when compared to compact and round islets in wild type embryos, the mutant islets were streaked along the pancreatic ducts (Fig. 10C), similar to mice lacking EGF receptor [6]. This phenotype is also very similar to that of zebrafish *wnt-5 *and *fz-2 *morphants, in which glucagon positive cells are scattered along the A-P axis while positioned at the periphery of *insulin*-positive cells (Fig. 5E,F).

To examine whether defects in endocrine cell migration during islet formation can cause streaked islets of *Wnt5a*^-/- ^embryos we used a previously described morphometry method [6]. Since insulin-positive cells have to detach and migrate away from the pancreatic ducts to form mature islets, one of the measures of islet cell migration is the degree to which insulin-positive cells remain in direct contact with the ductal epithelium. To assess migration of the endocrine cells in *Wnt5a *null embryos, we concentrated our analysis at E18.5, the latest time point at which we can assess separation of insulin positive cells from the ducts. Lethality at birth of *Wnt5a *knockout mice prohibits later analysis of pancreas development in these mice. When compared to wild-type embryos, the number of islets separated from the ducts was greatly reduced in the pancreata of *Wnt5a*^-/-^embryos (Fig. 10A). Only 15% of small islets and 6% of larger islets were separated from the ducts in the mutants, compared to 56% (P < 0.001) and 40% (P < 0.05) respectively in the wild type mice (Fig. 10B,C). Our results implicate that Wnt5a signaling is also important in the insulin-positive cell migration process during morphogenesis of the pancreas in higher vertebrates.

## Discussion

### A hypothetical role of Wnt signaling in early pancreatic development

The function of a *wnt-5/fz-2 *pathway in *insulin*-positive cell migration is most likely to provide the right environment and/or signals for *insulin*-positive cell migration during islet formation. Our time-lapse analysis of *fz-2 *MO-injected transgenic embryos shows that the *insulin*-positive cells still migrate, but their direction of migration is random indicating these cells have lost key positional information in the absence of the *wnt5/fz2 *signal (Fig. 1). In addition, our cell sorting analysis shows that *wnt-5 *RNA is expressed in a posterior-anterior gradient, potentially serving as a key step in establishing the positional information required by these migrating cells. Analysis of the receptor, *fz-2*, however, reveals a further complexity. *Fz-2 *RNA is detected in the cells immediately adjacent to the mis-migrating *insulin*-positive cells, suggesting that the function of wnt-5/fz-2 pathway activation in islet cell formation is a cell-non-autonomous and/or cell-cell instructive function in the transmission of this positional information (Fig. 2B). Interestingly, non-autonomous *fz*-mediated signaling has been observed during *Drosophila *development [51], but this process of information exchange does not require any cell migration movements or, apparently, any *wnt *ligand [52]. Further study is required to determine how the wnt-5/fz-2 signaling produces the right environment that allows normal *insulin*-positive cell migration during islet formation.

The activation of Wnt-5 signaling can change intracellular Ca^2+ ^concentration [26]. One hypothesized function of the Wnt/Ca^2+ ^pathway is to antagonize a second and simultaneously active Wnt/β-catenin pathway [27,28]. Wnt/β-catenin signaling is known to specify cell fate directly [2]. For example, reduction of maternal and zygotic *wnt-5 *activity using genetic approaches results in hyperdorsalized embryos [27]. Further investigation is required to determine whether wnt-5/fz-2 signaling provides the right environment and/or signals for normal insulin-positive cell migration by antagonizing the Wnt/β-catenin pathway or by another novel mechanism.

We observed an altered expression pattern of *pdx*-1 transcript in both *wnt-5 *and *fz-2 *morphant embryos. In our time-lapse imaging analysis, however, we observed that the initial specification of *insulin*-expressing cells was normal, and we observed no ectopic *insulin*-positive cells in spite of expanded *pdx*-1 expression in *fz-2 *or *wnt-5 *morphant embryos. Furthermore, the number of *insulin*-expressing cells is not increased in either *fz-2 *or *wnt-5 *morphant embryos even though these embryos displayed two or more insulin expression domains, sometimes with single cell patches, as observed in our time-lapse imaging analyses. These data suggest that the scattered *insulin*-expression is not due to ectopic islet formation. Future work will be required to detail the role (if any) for this expanded *pdx*-1 expression in abnormal *insulin*-positive cell migration.

### A role for *Wnt-5 *in pancreatic islet formation is conserved between mammals and teleosts

Vertebrate pancreatic development is described here in one of its simplest forms in zebrafish (represented by a single islet at 24 hours of development) and by a more complex vertebrate system in mouse. Certain processes of pancreatic islet formation in zebrafish and mouse appear to be incongruent. For example, sonic hedgehog (*shh*), which is expressed in the entire endoderm except for the pancreatic endoderm, was shown to be a negative regulator of the adoption of pancreatic endoderm fate in mice [29,30]. In zebrafish, however, *shh *and other *hh *proteins are not expressed in the endoderm [31]. In addition, ectopic expression of *shh *induces a pancreatic cell fate rather than represses it, a result opposite to what was observed in mammalian models [31,32]. Despite these differences, the initial specification of *insulin*-expressing cells occurs before an islet is formed in both animals, and these *insulin*-positive cells migrate and form an islet at a distance from where they are initially specified. Even though a comparable structure to a duct does not exist in zebrafish at the stage when islet formation occurs, previous studies in mouse and our time-lapse imaging analysis show that this migration process of islet formation is similarly required to form normal islets in both mouse and zebrafish. The final disorganized islet phenotypes were observed as a result of abnormal migration of *insulin*-positive cells in both zebrafish and mouse embryos with disrupted Wnt5 signaling. These results argue that Wnt5 signaling is required in pancreatic islet formation of both mouse and zebrafish, a role most likely shared throughout the vertebrate lineage.

### Fz-2 is a putative receptor for Wnt-5 in pancreatic islet formation

Our results suggest that *wnt*-5 is upstream of *fz*-2 in the same genetic pathway and show that Fz-2 functions as a receptor for Wnt-5 when placed in the right environment, as assayed using the *Xenopus *secondary axis assay system. In mouse, *fz*-2 and *wnt*-5 transcripts are expressed at the highest level among different *wnt*s and *fzs *in the developing mouse pancreas, providing evidence that *wnt-*5 and *fz*-2 are expressed at the right place and time to be involved in islet formation as a functional ligand and receptor pair [3]. In zebrafish, early *fz*-2 expression is observed during somitogenesis. This expression pattern coincides with the timing and location of insulin cell migration, indicating that *fz*-2 is expressed at the right time and place to be involved in normal insulin-positive cell migration in zebrafish. Although *wnt*-5, *wnt*-11 and *wnt*-8 are expressed at the 14-somite stage, only *wnt*-5 and *wnt-11 *transcripts are detected at the location close to the presumptive pancreatic endoderm marked by *pdx*-1. *wnt-5 *is not expressed in the endoderm, but it is expressed in adjacent mesoderm close to the posterior boundary of early *pdx*-1 expression. *wnt-5 *proteins may be secreted from the mesoderm and signal to adjacent endoderm. Furthermore, our data show that *wnt*-11 morphant embryos have a normal pancreatic islet formation, whereas *wnt-5 *morphant embryos have similar pancreatic islet formation defects as *fz-2 *morphant embryos placing *wnt-5 *as the best candidate ligand for *fz-2 *in pancreatic islet formation.

### Wnt-5 signaling has a specific role in pancreatic islet formation that is separable from other Wnt signaling events in embryogenesis

In embryos with defects in body axis elongation, some significant secondary effects on other organizing centers may occur. For example, zebrafish *wnt*-5 [20], *wnt*-8 [19], *wnt*-11 [33] and *knypek *[34] morphant or mutant embryos display undulating notochord, reduced body length and a number of other less apparent abnormalities. These defects could conceivably result in abnormal pancreatic islet formation. However, *wnt-8 *and *wnt-11 *morphant embryos display normal islet formation despite body elongation defects. This supports a specific role of Wnt-5 signaling in pancreatic islet formation that is not secondary to body elongation defects. In addition, injection of *wn*t-5 and *fz*-2 mRNA ameliorates the pancreatic islet formation defects in *wnt-5 *and *fz-2 *morphant embryos, respectively, but not the body elongation defects under the same conditions. These results strongly suggest that pancreatic islet formation is at least partially independent of the body axis elongation process. In addition, the initial specification of *insulin*-positive cells occurs at the right place and time in zebrafish embryos. The congregation of *insulin*-positive cells occurs normally, and glucagon cells surround the insulin-positive cells in mouse *Wnt5a*^-/-^knockout embryos and *wnt-5 *and *fz-2 *morphant zebrafish embryos. Finally, the development of a grossly normal pancreas in *Wnt5a*^-/- ^mouse knockout embryos, and the subsequent defect in islet formation, emphasizes the possible function of Wnt5 signaling during later stages of pancreatic islet morphogenesis.

## Conclusion

We examined a role of *Wnt-5 *signaling in pancreatic islet morphogenesis in zebrafish and mouse embryos. Time-lapse imaging of *insulin*-positive cells using transgenic zebrafish embryos revealed that gradual formation of a single islet in zebrafish is a result of an active cell migration process. Here we demonstrate that *wnt-5 *and *fz-2 *morphant embryos display defects in *insulin*-positive cell migration providing evidence for a role of Wnt5 signaling in this process. Comparative analyses of pancreatic tissue from *Wnt5a *knockout mice show that endocrine cells are specified normally within the primitive pancreatic epithelium. However, these *insulin*-positive cells fail to migrate from the ducts and form fragmented islets around ducts with *glucagon*-expressing cells positioned at the periphery. This argues that *Wnt5a *is required for *insulin*-positive cell migration, but not local organization within a forming islet in both mouse and zebrafish. Taken together, these results provide evidence that *wnt*-5/*fz*-2 signaling is required for the migration of *insulin*-positive cells during pancreatic development, and that this function is conserved. This study will help us to understand better the role of Wnt signaling during vertebrate pancreatic development and broaden our understanding of a role of Wnt signaling in pancreatic diseases. Wnt signaling pathways have been implicated in the pathogenesis of cancer [35], regulation of adipogenesis and insulin secretion [36,37]. *Wnt5b*, for example, has recently been identified as a candidate gene for conferring susceptibility to type 2 diabetes [38]. *Wnt5a *may inhibit the canonical Wnt pathway by promoting β-catenin degradation [28]. Interestingly, dysregulation of β-catenin has been identified in various forms of malignancy, including pancreatic tumors [39]. Our study reports a new and conserved role of Wnt-5 signaling in pancreatic islet formation and provides an example of how Wnt signaling functions in organogenesis.

## Methods

### Synthesis and microinjection of mRNAs

Wnt-5 ORF cDNA was amplified from a gastrula stage library and subcloned into pT3TS [40]. The modified wnt-5* construct was made using a degenerate 5' primer designed to have a mismatch with the published wnt-5 MO (5'-ATG GAC GTA CGT ATG AAT CAG GGT CAC CTA CTT CTG GCA G-3'). The degenerate PCR fragment was subsequently subcloned into pT3TS vector at *SpeI *site. To synthesize sense *wnt*-5 mRNA, the *wnt*-5-T3TS construct was digested with *XbaI *and transcribed using T3 mMessage mMachine kit (Ambion). Modified *wnt*-5 mRNA (4 pg) was injected for the *wnt-*5 rescue experiment.

*Fz*-2 ORF cDNA was amplified from the gastrula library and subcloned into pT3TS. *Fz*-2 construct was cut with *XbaI *and transcribed with T3 mMessage mMachine kit (Ambion). For *Xenopus *assay, 500 pg of *fz*-2 mRNA was injected into two ventral blastomeres of 4-cell stage *Xenopus *embryos. For the *fz*-2 MO specificity experiment and *wnt*-5 and *fz*-2 epistasis experiment, 25 pg of *fz-*2 mRNA was injected.

### Microinjection of MOs

We injected 2.5 ng of the previously described *wnt*-5 MO for the *wnt*-5 and *fz*-2 genetic interaction experiment and 8 ng of the same *wnt*-5 MO for all other experiments [17]. We also used 5 ng of wnt-5 MO (5'-TGTTTATTTCCTCACCATTCTTCCG-3') targeting the 3' end of the exon-intron junction of exon 3. As a control, 8 ng of a five-base mismatch wnt-5 MO (5'-GTCGTTGCTTCTTTCACACTTCCAT-3') was injected.

For the analysis of different pancreatic markers, we injected 5 ng of *fz*-2 MO mix (2.5 ng of *fz *MO-1 and 2.5 ng of MO-2) as described [14]. As a control, 5 ng of a five-base mismatch fz-2 morpholino (5'-CCTCCATAGTCACGATAAGTTCGGC-3') was injected. For the *wnt-5/fz-2 *genetic interaction experiment, we injected 2 ng of *fz*-2 MO mix (1 ng of *fz*-2 MO-1 and 1 ng of *fz*-2 MO-2). For the FZ-2 morphant rescue experiment, we used 2 ng *fz*-2 MO-1.

We injected 2–4 ng of the previously described wnt-11 MO to generate WNT-11 morphant embryos [17]. Two specific MOs targeting WNT-8 have been previously described [16,19]. Embryos were injected with 1–8 ng of *wnt*-8 MO1, 1–5 ng of *wnt-*8 MO2 or 2 ng *wnt*-8 MO mix (1 ng of *wnt-*8 MO1 and 1 ng of *wnt-*8 MO2).

All three groups of embryos were allowed to develop for 24 hours and subsequently analyzed for pancreatic markers. Microinjections were performed at the one-cell stage as described [40].

### *In situ *hybridization

Single color *in situ *hybridization was performed as described [41]. Double color *in situ *hybridization was performed as described [42]. *Fz*-2 antisense riboprobe was synthesized as described [14]. To synthesize *wnt*-5 probe, *wnt*-5 ORF cDNA was sub-cloned into 4-TOPO vector (Invitrogen) and subsequently cut with *NsiI *and transcribed with T3 RNA polymerase. The following probes were used: *pdx-*1, *insulin *[43], *glucagon*, *somatostatin *[11], *islet*-1 [44], *fspondin*-2b [45], *sox*-17 [46], *fox-A*2 [47], *gata*-6 [48], *mixer *[35], *ceruloplasmin *[49] and *carboxypeptidase A *[32]. The *mixer *probe construct was synthesized by sub-cloning a 1 kb PCR fragment into the 4-TOPO vector. To synthesize the probe, DNA was cut with *NotI *and transcribed with T3. The *fox-A2 *probe construct was synthesized by sub-cloning a 1 kb PCR fragment of *fox-A2 *into the 4-TOPO vector. To synthesize the probe, DNA was digested with *NotI *and transcribed with T3. The *carboxypeptidase A *probe construct was synthesized by subcloning a 500 bp PCR fragment into the TOPO vector. To synthesize the probe, DNA was digested with *NotI *and transcribed with T3. The *gata-6 *probe was synthesized by digesting an EST clone obtained from ZFIN (cb 603) with *Sal1 *and transcribing with SP6.

### RNA microinjection into *Xenopus *embryos

Wild-type zebrafish *wnt*-5 mRNA (250 pg) and 500 ng of zebrafish *fz*-2 RNA was injected alone or in conjunction into the two ventral blastomeres of the 4-cell stage *Xenopus *embryos (total of 500 pg wnt-5 RNA and 1 ng of fz-2 RNA). As a control, 500 ng of GFP RNA was injected alone or together with *fz*-2 RNA. The injected embryos were scored for double axes at the tailbud stage. Injections were performed as described [40].

### Time-lapse imaging

The 14 somite stage embryos were dechorionated and embedded in 0.8% low melting point agarose with 0.05% 3-aminobenzoic acid ethyl ester in a home made holding chamber. To prevent drying, the embedded embryo was covered with mineral oil and then overlaid with a cover slip. The images were captured every minute for 4–6 hours using a digital camera (Hamamastu, C4742-95) attached to a compound microscope with automated base (Zeiss) and OpenLab program.

### Cell dissociation and FACS analysis

Insulin:GFP transgenic zebrafish embryos at the 20 som stage were dechorionated and homogenized in cold 1X Danieau/ 15% fetal bovine serum (FBS) solution using a tissue grinder. Homogenized embryos were washed twice with cold 1X Dan/15% FBS and incubated in 1X Danieau/ 15% FBS containing 10 mg/ml collagenase/dispase (Roche, #10269638001), 10 mg/ml trypsin and 10 unit/ml DNase 1 at 30°C for one hour with gentle agitation. Dissociated cells were subsequently washed twice, filtered through a cell strainer and finally suspended in 1X Dan/15% FBS supplemented with 1 μg/ml DAPI (Roche, #10236276001: to label dead cells and other debris). Cells were sorted using a cell sorter (BD science), collected in 100% FBS, and processed for subsequent total RNA isolation using an RNAqueous-4PCR kit (Ambion, #1914). For subsequent RT-PCR, cDNA was synthesized using Powerscript Reverse Transcriptase (BD science, #8460). The following primers were used. EF1-a: TCACCCTGGGAGTGAAACAGC, ACTTGCAGGCGATGTGAGCAG, insulin: CCATATCCACCATTCCTCG, CGGAGAGCATTAAGGCCTG, fz-2 set#1: ATGCAGGCGAGTGGAAGTG, CTTATAGCTGAGGTAGGCTG, fz-2 set#2: TGGCGTCTGGACAGCATC, TCCTGGTCTGTGAAGAACAT, wnt-5: ATGGATGTGAGAATGAACCAA, CTACTTGCACACAAACTGGTC.

### Genotyping of zebrafish *ppt*^*hi*1780*b *^allele

Zebrafish embryos injected with low dose exon-intron blocking wnt-5 MO were raised up to 3 days. The heads of these embryos were excised and frozen in -80°C for further genotyping and the bodies were further processed for insulin expression using *in situ *hybridization. After the insulin expression pattern analysis, genomic DNA of 20 embryos with normal and abnormal insulin expression pattern was isolated from the frozen head tissue and genotyped as described [21].

### Genotyping of mice

Genomic DNA was isolated from tails or yolk sacs by standard methods [50] and amplified by PCR using the following primers: W1 (5'-GAC TTC CTG GTG AGG GTG CGT G-3'), W2 (5'-GGA GAA TGG GCA CAC AGA ATC AAC-3'), and W3 (5'-GGG AGC CGG TTG GCG CTA CCG GTG G-3'). The PCR settings were: 30 cycles, 94°C for 30 seconds, 65°C for 30 seconds, 72°C for 1 minute. A 360 bp band identified a wild-type allele, while a 200 bp band identified the mutant allele.

### Histological analysis

Embryos used in this study were obtained by intercrossing *Wnt5*^+/- ^mice [24]. Plug date was considered E0.5. The wild type (N = 5) and *Wnt5a*^-/- ^(N = 8) embryos were harvested between E16.5-E18.5. The pancreatic tissue was dissected from the embryos, fixed in 4% paraformaldehyde, washed in PBS, dehydrated through alcohols, embedded in paraffin and cut into 7–9 μm thick sections.

### Immunohistochemistry

Immunohistostaining to detect the islets of Langerhans was done using commercially available mouse monoclonal anti-insulin antibodies (SIGMA I-2018) for β-cells, and anti-glucagon (G-2654) antibodies for α-cells as previously described [51]. The sections were rehydrated, incubated in methanol containing 1% H2O2, and washed in PBS. The sections were then incubated with primary antibodies (concentration 1:500) at 4°C overnight. After washing the slides in PBS, the sections were incubated with biotinylated mouse IgG at 1:500 (Vector Laboratories) at room temperature for one hour. In order to detect the staining, the slides were incubated in Vectastain reagent for 30 minutes, followed by the diaminobenzidine detection method. The slides were then background stained by dipping them in hematoxylin 2 for 5 seconds, washed and cover slipped.

### Morphometry

We defined small islets as insulin-positive aggregates 25–70 μm in diameter and large islets as aggregates greater than 70 μm in diameter. A total of 31 islets from 3 wild type embryos and 56 islets from 5 *Wnt5a*^-/- ^embryos were counted and classified as being either associated with ductal tissue or not associated. Islets were defined as associated with ductal tissue if insulin-positive cells were in direct contact with duct epithelium [6]. 16 WT and 38 knockout islets 20–70 μm in size, and 15 WT and 18 knockout islets over 70 μm in size, were counted and classified.

### Statistical analysis

The statistical significance between the groups was determined by the Student's T-test for zebrafish experiments and by a Chi-Square test for the mouse experiment.

## Authors' contributions

SCE conceived and designed this study. HJK conducted zebrafish and Xenopus experiments, analyzed results and drafted the manuscript. SS made the initial observation of defects in pancreatic islets of fz-2 morphants. JRS and AP performed immunohistochemistry of pancreatic islets from wild-type and wnt-5a KO mice, and JJ analyzed the results. JRS conducted islet morphometric analyses. SCE, AP and SL supervised the study. SCE and AP edited the manuscript. All authors read and approved the final manuscript.

## Supplementary Material

Additional File 1 **(QuickTime format) WT movie**. Figure 1C–H panels were taken from this movie at the indicated time points. GFP-positive cells are first observed around the 14 somite stage as bilateral patches in insulin:GFP transgenic embryos. These cells divide and actively migrate to the posterior. The net movement towards the midline is an indirect result of convergent-extension since active migration towards the midline is not observed. By the 24 hpf stage, GFP-positive cells are coalesced into a single islet in the posterior and midline relative to the starting position. Click here for file

Additional File 2 **Two color time-lapse imaging shows that GFP-positive cells migrate relative to the neighboring cells**. (A) GFP-positive cells are first visible posterior to rhodamine-labeled cells as bilateral lows of cells. (B-G) GFP-positive cells migrate posteriorly and medially, whereas rhodamine-labeled cells do not change their relative position. (H) At 24 hpf, all GFP-positive cells coalesced into a single islet, but the rhodamine-labeled cells remain separated at their original position. Arrows: rhodamine-labeled cells; t: time (minutes). Click here for file

Additional File 3 **(QuickTime format) FZ-2 morphant movie**. Figure 1I–N panels were taken from this movie at the indicated time points. As also noted in non-injected insulin:GFP transgenic embryos, GFP-positive cells are first observed around the 14 somite stage as bilateral patches, and these cells divide normally in *fz-2 *MO-injected embryos. However, the uniform migration towards the posterior observed in the non-injected transgenic embryo is not observed in *fz-2 *MO-injected embryos. Instead, the polarity of cell migration is entirely lost, with no net movement along the anterior-posterior axis observed for these cells (see text). Click here for file

Additional File 4 **Genotyping of embryos with normal and abnormal insulin expression**. (A) 90% of embryos with abnormal insulin expression are carriers of *ppt*^*hi*1780*b*^. Lane 4 and lane 15 represent wild-type embryos with abnormal insulin expression. (B) Control PCR for exon 1 of *wnt-5*, N = water only control. (C) None of the embryos with normal insulin expression are carriers of *ppt*^*hi*1780*b*^. (D) Control PCR for exon 1 of *wnt-5*, N = water only control. Click here for file
