# Supplementary figures and images for: Wnt5 signaling in vertebrate pancreas development
Source: BMC Biol. 2005 Oct 24;3:23. doi: 10.1186/1741-7007-3-23 (PMC1276788; doi:10.1186/1741-7007-3-23)

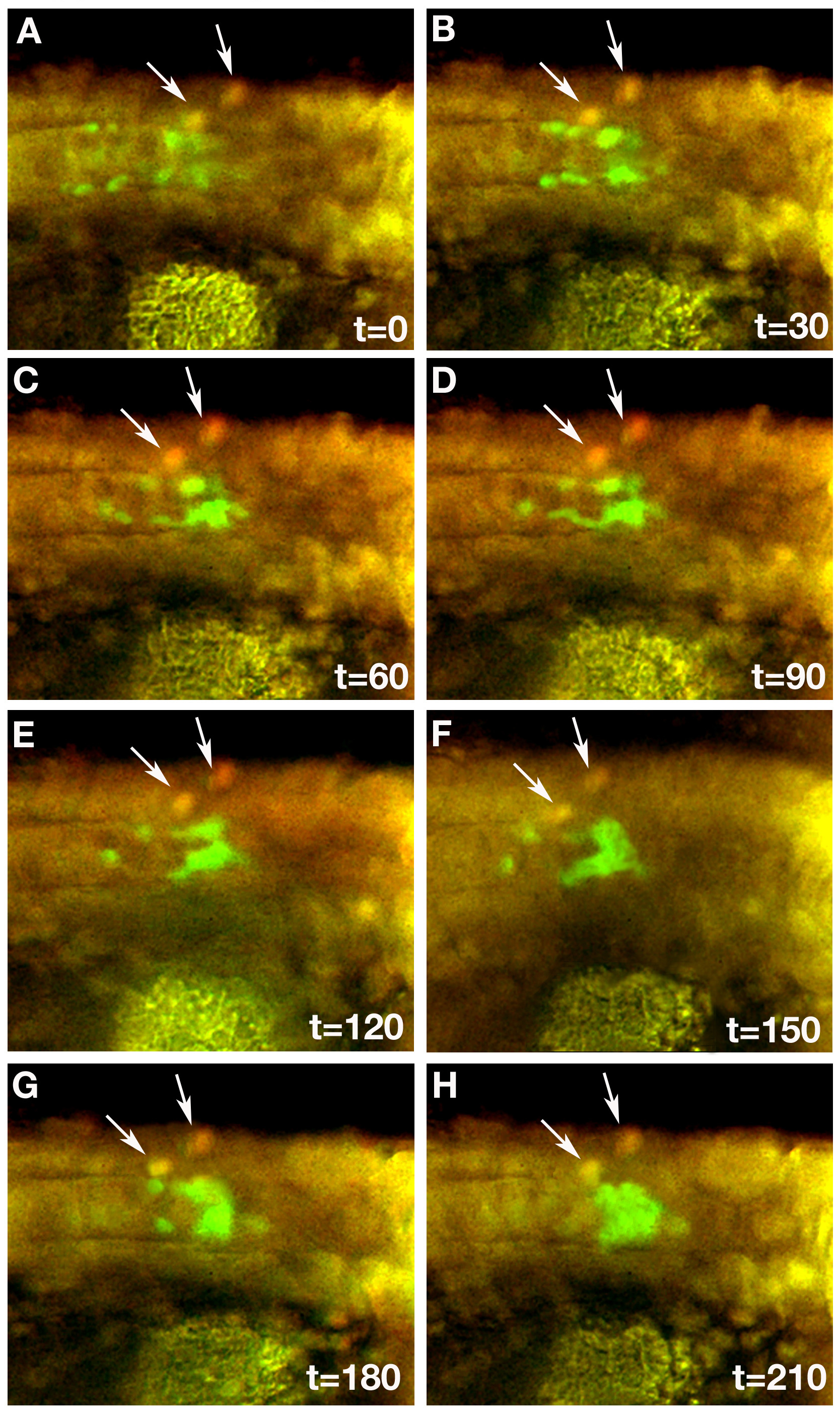

Supplement: Additional File 2 — Two color time-lapse imaging shows that GFP-positive cells migrate relative to the neighboring cells. (A) GFP-positive cells are first visible posterior to rhodamine-labeled cells as bilateral lows of cells. (B-G) GFP-positive cells migrate posteriorly and medially, whereas rhodamine-labeled cells do not change their relative position. (H) At 24 hpf, all GFP-positive cells coalesced into a single islet, but the rhodamine-labeled cells remain separated at their original position. Arrows: rhodamine-labeled cells; t: time (minutes). [file 1741-7007-3-23-S2.jpeg]

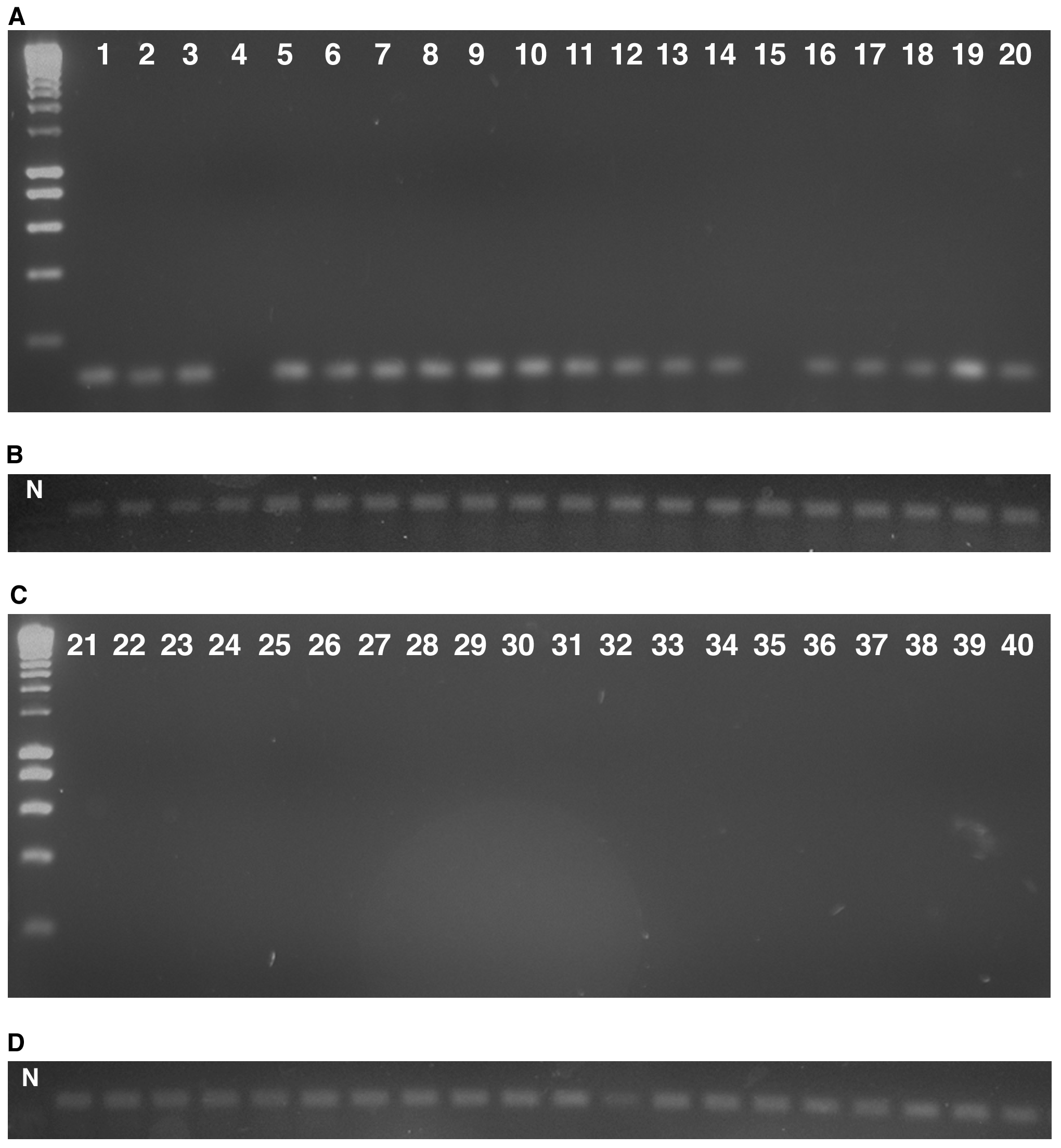

Supplement: Additional File 4 — Genotyping of embryos with normal and abnormal insulin expression. (A) 90% of embryos with abnormal insulin expression are carriers of ppthi1780b. Lane 4 and lane 15 represent wild-type embryos with abnormal insulin expression. (B) Control PCR for exon 1 of wnt-5, N = water only control. (C) None of the embryos with normal insulin expression are carriers of ppthi1780b. (D) Control PCR for exon 1 of wnt-5, N = water only control. [file 1741-7007-3-23-S4.jpeg]
